# The Doctor as a Peacemaker

**Published:** 1898-08

**Authors:** J. O. Scott

**Affiliations:** Sherman, Texas


### TEXAS MEDICAL JOURNAL.

ESTABLISHED JULY, 1885.

PUBLISHED MONTHLY.—SUBSCRIPTION $1.00 A YEAR.

VOL. XIV. AUSTIN, AUGUST, 1898. No. 2.

### Original Contributions.

For the Texas Medical Journal

### The Doctor as a Peacemaker.

BY J. O. SCOTT, M. D., SHERMAN, TEXAS.

[Read at Fort Worth meeting North Texas Medical Society.]

About one hundred and fifty miles north of Jerusalem there spreads a beautiful country, picturesque and enchanting; studded with green clad mountains and crystal lakes, the head-waters of sparkling streams, that meander through fertile valleys. In this lovely land, where God's own chosen people lived centuries ago, there is a lake called Galilee—near by a mountain, upon the side of the mountain facing the glassy waters of this pellucid lake our Saviour called his chosen twelve, and gave them and us the promise of blessings, more precious than all the gold of Ophir or the diamonds that sparkle beneath the waters of Golconda.

There are blessings for the pure in heart, the meek, the merciful, but the blessing to the peacemaker is the most gracious promise from God to man. There is no expression in all divinity so comforting as the precious, undying words of the immaculate Savior, "Blessed are the peacemakers; for they shall be called the children of God." Of all on earth there are none so dear to us as our offspring,—What a blessing to be reckoned a child of the great Jehovah.

It was a bitter cold winter night—the snow was falling fast when we left the cottage, assuring the parents that the child had passed the danger line and was resting comfortably.

Later the child became a little restless. A busy-body influenced the frightened parents to send for another physician near by. He came and sent for us; in our presence he assured the parents that the little one was in no immediate danger, and that the medicine administered had had the desired effect. Then he excused himself. The room shone with radiant brightness as if lit by angel lamps. It was the reflection of benevolence, of kindness, of love, of honor, of purity, of nobleness—bright sparks gathered from the etherial throne. Like the corruscations of a streaming comet, they sparkled and shone, making celestial brightness of stygian darkness.

While waiting and watching after our physician friend had left, we read the following, which some one had written on the fly leaf of an old book, lying on the table under our gaze: "In the valley of Virginia, that beautiful valley famous for its loveliness the world over, was fought by Stonewall Jackson the battle of Winchester. To the west of the town there is a high ridge on which Genl. Banks' troops were massed in hostile array. The frowning fortress bristled with small arms and was fortified with huge ordnance sending grim death and destruction with every volley. When Genl. Rich Taylor rode up, Jackson pointing to the smoke-enveloped ridge said: 'You *must* carry it.'"

In a few moments Taylor with his Louisiana Tigers were climbing the slope, and in a short time the crescent flag, the gilded banner of the brave Louisianians was waving on the summit rejoicing in glorious victory. That night after the battle, while the stars in heaven were craped in mourning for the scene of death and suffering, during the silent darkness, Genl. Jackson came to Taylor's camp-fire. Genl. Taylor says sometimes he conversed and often sat motionless as if praying to the great God of battles for aid, or thanking him for the brilliant victory just won. During the long hours of this night of sorrow and rejoicing, Taylor informed Jackson that Genl. Winder was dissatisfied that a furlough had not been granted to him and thought of resigning his commission. Taylor thought Winder's services too valuable to be lost to his country and suggested to Genl. Jackson to see Winder. The next day Genl. Winder informed Genl. Taylor that Genl. Jackson had called to see him and that he had determined it best for his country not to resign. In a few days Genl. Winder fell garlanded with everlasting renown at the battle of Cedar Mountain while gallantly leading the famous Stonewall Brigade to a brilliant victory.

A few months later, Stonewall Jackson was shrouded in immortal fame on the bloody field of Chancellorsville. There is a camp fire on "fame's eternal camping ground." Jackson is there rejoicing with the crown of glory of the Christian warrior. Winder, with a halo of resplendent grandeur round about him, is seated by the side of his great leader. Taylor too has been borne on angel wings to the blissful paradise of the patriot brave, and the great Jehovah has decked his brow with a brilliant diadem, studded with the glittering words of inspiration, "blessed are the peacemakers for they shall be called the children of God."

It was a beautiful summer's night; the pale silvery moon shone with its mellow light through the latticed windows of our bed room. All the stars in heaven seemed to smile in delight in the radiant light, and whisper "peace, peace, peace." We are thinking of our patient in a distant block, when the door-bell rang. After hastily dressing we found at the door a physician and another visitor, the husband of a lady patient. During the night our patient had a severe chill. Her husband becoming alarmed went for the consulting physician—not knowing the courtesy from one doctor to another. The consultant would have visited that lady under no circumstances unless with us, or in our absence, in an emergency. While we were walking along silently to the house of the sick lady, as the revolving moon shone on his generous and noble face, we could but admire and love him for his sterling virtues, his high sense of honor, refined sentiment and intuitive knowledge of duty and right.

The next day we were reading Homer's Iliad, and when we read the words,

> "Patrochlus loved of all our martial train,
> Thy form so pleasing and thy heart so kind,"

we thought of him. At the end of the chapter we find this we had written:

"We see on the shores of Troy the mighty Achilles sulking in his tent angry for fancied wrongs, Patrochlus his best friend, —the surgeon—pacifies his high resentment to the towering Agamemnon the leader of the Grecian host. Again the sword of Achilles is unsheathed, Hector is slain by his mighty falchon, and Troy falls by his god-like prowess. The mild rays of peace burst through the clouds of horrid war. Upon the crimson field of far-famed Troy, where the blood of heroes dyed the waters of the Simois, Achilles and Patrochlus fell clasped in glory's arms, renowned for heroic deeds through all the rolling centuries of the past."

Near where the loud-roaring waves sweep the seaside, there is a monument to Achilles; upon the wave-washed crowning stone the passer-by can read: "Here lies the great Achilles, victor of Hector slain." Near by, is the shaft of Patrochlus, the warrior surgeon, the pacificator of Achilles to Agamemnon. On the glittering surface of the marble shines with refulgent beauty, the heaven-born words: "Blessed are the peacemakers for they shall be called the children of God."

When a school boy in Kentucky we boarded at the house of a physician in a small village. There was another old doctor in the village, his life-long professional rival. These two physicians had not recognized each other in public, or private, for a score of years. During the year we were sojourning with Dr. Mitchell, his professional rival was sued for mal-practice. Dr. Mitchell, who had been sent for to see the case, saw the surgical work was right, and so informed the lawyer for the prosecution. The case was dismissed to the great mental relief of the rival.

A short time after that occurrence, the beautiful and lovely daughter of Dr. Mitchell, the belle of the village, the idol of her father's heart, was very sick. The physician who was sent for from Lexington was unable to attend her. It was the cold Friday in January, 1848; no one was leaving the snow-covered houses unless compelled to by dire necessity, or some physician in his business of relieving distress and suffering. While the household, old and young, were comfortably seated by a hot blazing fire whiling away the tedious hours drinking old crab-apple cider, eating fine, delicious pippin apples and scaly-bark hickory-nuts, a servant knocked at the door. He was the bearer of a note to Dr. Mitchell from his professional rival, who was seated in his buggy at the front gate. He desired to know if his services to the sick young lady would be acceptable. The forgiving words, the embraces, the reconciliation of these two old gray-headed doctors, on the verge of eternity, would have melted a heart of adamante. Thoughtless boy as we were, as with the rest of the family, tears came to our eyes. A young physician, a former student of Dr. Mitchell, had, in a casual conversation, informed the rival that he had heard Dr. Mitchell say that he had no ill will to any one on earth, especially his competitor in the village. The young doctor happened to be in the house at the time, a witness to the reconciliation to which he was the mediator. Dr. Mitchell invited his competitor and visitor to see and prescribe for his sick daughter. After examining the young lady and pronouncing her condition not dangerous, they all sat down to a dinner after the Kentucky style of hospitality. Upon the lavishly spread dining table of this hospitable mansion there was the epicurian's delight, the three-year-old boiled ham; a fat turkey; a choice roasted saddle of southdown mutton, juicy and savory smoking fresh from the embers; Baltimore oysters, fried, stewed and raw; fish salads; all kinds of vegetables; fruits, cakes and cream; all enjoying the feast and happy over the reconciliation. After this sumptuous repast the patriarch of the family, the pious old village physician, called for his family Bible and all joined in family worship. The good book was extended to the visiting physician by the host. The guest, who was also a devout Christian, opened the old time-worn family book of inspiration, and read from the fifth chapter of Matthew; when he came to the fifth verse, his fading blue eyes gazed kindly and gratefully upon the young doctor and the tears streaming over his cheeks, with tremulous voice he read the most comforting words to the virtuous in the Bible: "Blessed are the peacemakers for they shall be called the children of God."

It was our good fortune to spend the winter of 1859-60 on Galveston bay at the hospitable home of Col. Jas. Morgan. At daylight on the morning of our arrival we gazed across the bay and it seemed we were in an earthly paradise—an enchanted land. The waters were filled with geese, ducks and swans, and on the green undulating prairies in the distance we could observe small groups of antelopes grazing. All varieties of fish filled the waters of the contiguous shores, and from our window we could see the fishermen with their nets drawing to the shore the finny tribe of all varieties and sizes. Far in the distance across the white-capped waves the spires of Galveston city, "the bride of the seas," glittered and sparkled the refulgent rays of the morning sun. Through the courtesy of Col. Morgan, we were invited to dine with Dr. Ashbel Smith, who has since that time been the honored president of the Texas State Medical Society and has immortalized his name, as a hero of the 2d Texas Regiment, leading his command to glorious victory and renown at Shiloh, Vicksburg and other battles. His home was a lovely cottage by the sea, with the "bonny brier bush" creeping over the latticed porch. Under its hospitable roof had been entertained by the scholarly and generous host Com. Vanderbilt, Audubon, the botanist, Sam Houston, Sidney Sherman, Rusk, Dr. Anson Jones, Burleson, Lamar, and many other distinguished Texans. The doctor was a bachelor. His faithful rifle rested on the horns of some antlered monarch of the waste that had fallen under its never-erring fire; shells and nautical curio were scattered hither and thither like the playthings of a child. Piled around on tables and shelves were rare books he had gathered while Minister to France and Great Britain under Houston. We saw hanging in his studio his commission as first surgeon-general of the Republic of Texas. When the afternoon dinner was announced we were ready, for the salt air was invigorating and sharpened the appetite keenly. On the profusely-laden table were canvas-back duck, red fish chowder, trout, wild turkey and venison, oysters cooked in every style, salads, vegetables, and many delicacies. After this bountiful repast, in company with the doctor, we started for a visit to a small island opposite, where there was a magnificent view of the surrounding bay country. Pointing to Boliver Point the doctor's eyes sparkled with the fire of youth, his cheeks crimsoned and he became eloquent. There is the spot where Dr. Jas. Long built his fort. Years ago he was a surgeon in Jackson's army. In 1819 he left Natchez, and arriving at Nacogdoches, he was the first to plant the lone star flag—the banner of the Republic, on Texas soil and proclaim it a free and independent State. At that fort his heroic and faithful wife spent eight long months alone with her two children and servant, every hour expecting the return of her chivalrous husband from his disastrous expedition against the Mexicans. She died broken hearted when she heard of his tragic death at Goliad. He was murdered by the Mexicans after surrendering. Sailors tell us that the spot is haunted and they can see a tall white woman, covered with the foam of the sea walking up and down the beach, on stormy nights, pulling her dishevelled hair, beating her bony breast and wringing her long fleshless arms.

At the storming of San Antonio, Dr. Grant, an English physician, hand to hand with Deaf Smith, Ben Milam, Johnson (?) and others, displayed great heroism. A short while after this, February, 1836, he with Col. Johnson led the first expedition against the Mexicans. Before the fall of the Alamo in a battle near San Patricio he and all his comrades fell while battling bravely for the cause of freedom. As Genl. Warren at Bunker Hill and Genl. Mercer at Princeton, both doctors, shed their life blood for American independence, so these Texan heroes and physicians were immolated on the altar of liberty-martyrs for Texan independence.

Beyond the beautiful island facing his home, he said Lafitte's vessels anchored in those glassy waters after capturing Spanish galleons laden with rich cargoes of gold, silver and precious stone. Farther up the bay he pointed to us the historic live oak tree under whose shade Dr. Ewing gave surgical attention to Genl. Houston's wounded ankle. In the far distance our eyes were feasted with the lovely seaside home, where resided the accomplished widow and lovely daughter of Dr. Anson Jones—the last president of the Republic, under whom Dr. Ashbel Smith was Secretary of State. The "old man eloquent" informed us that Drs. Archer, Jones, Brown, Miller, Moore and Stewart fought gallantly for Texas independence; and that Dr. Wm. Motley, an aid to Genl. Rusk at San Jacinto in front of Burleson's Regiment met death and glory, bravely leading the Texans in the charge on the Mexican battery. That night we returned to New Washington, the hospitable home of Col. Morgan. We tried to entertain the Colonel, repeating Dr. Smith's instructive and agreeable conversation. Said Col. Morgan: "Smith is too modest to tell you anything of himself. He is the father of the University of Texas; he is a peacemaker with his neighbors and friends—that he had smoothed the animosities between Genls. Houston and Sidney Sherman. That the difficulty between Albert Sidney Johnson and Sam Houston, had become reconciled by his kind and brotherly mediation; that if he could have been on the ground a few moments sooner, Felix Houston never would have had wounded in a duel Genl. Johnston."

When speaking of his distinguished neighbor, Col. Morgan, the commodore under the Lone Star Flag of the republic, became enthused, ending his speech thus: "Dr. Ashbel Smith is a generous, noble, kind hearted and chivalric gentleman, a pacificator. Upon his monument under the name of Ashbel Smith I would chisel, 'Blessed are the peacemakers, for they shall be called the children of God.'"

All along the journey of life, the good old doctor, the kind old doctor, the faithful old doctor, the virtuous old doctor, the family physician is a peacemaker. He is the quieter of domestic broils, of public disturbances, neighborhood feuds and professional differences.

Beyond the blue vault of the ethereal skies, high up over the Milky-way, far away from starry Orion, the weeping Pleiades and the star of the North, the marriner's shining star that forever sheds its radiant light above the frozen sea—in the farthest heavens, farther on from where the blushing Venus eyes the mail-clad Mars, marching with proud martial step to the music of the revolving orbs, in the great highway of the imperial luminaries, there is the last resting place of the spirits of the illustrious, generous, noble and brave men, who have fought the battle of life, alleviating the sufferings of others. We need not pause to mark their names on marble or granite, or to tell the people of all their noble deeds with statues of brass or bronze; their fame has gone before them.

In the starry kingdom of the nether skies, there is a sphere revolving, from the vapory surface of whose happy shores, like Neptune from the seas, proudly arises in majestic grandeur, a monument of the ruby, the sapphire, and the emerald. Upon its high wrought surface is carved in brilliant letters, the million names of our co-laborers, each name encircled with a wreath of olive.

Like the statue of Memnon, in the far off east, at early matins, the hour of prayer, in sweet cadence are heard the melodious words: "Blessed are the peacemakers, for they shall be called the children of God."

When the crimson rays of the rising sun kisses the jeweled surface of that obelisk, the fading stars of the morning will shout for joy, and all the angelic hosts of heaven will sing in jubilee, "Blessed are the peacemakers, for they shall be called the children of God."

